# A Ubiquitin-Binding Domain that Binds a Structural Fold Distinct from that of Ubiquitin

**DOI:** 10.1016/j.str.2019.05.003

**Published:** 2019-08-06

**Authors:** Michael Lim, Joseph A. Newman, Hannah L. Williams, Laura Masino, Hazel Aitkenhead, Angeline E. Gravard, Opher Gileadi, Jesper Q. Svejstrup

**Affiliations:** 1 Mechanisms of Transcription Laboratory, The Francis Crick Institute, 1 Midland Road, London NW1 1AT, UK; 2 Structural Genomics Consortium and Target Discovery Institute, Nuffield Department of Clinical Medicine, University of Oxford, Oxford OX3 7DQ, UK; 3 Structural Biology Science Technology Platform, The Francis Crick Institute, 1 Midland Road, London NW1 1AT, UK

**Keywords:** ubiquitin, ubiquitin-binding domain, CUE domain, UBA domain, KAP1, TRIM28, TIF1β, SMARCAD1

## Abstract

Ubiquitylation, the posttranslational linkage of ubiquitin moieties to lysines in target proteins, helps regulate a myriad of biological processes. Ubiquitin, and sometimes ubiquitin-homology domains, are recognized by ubiquitin-binding domains, including CUE domains. CUE domains are thus generally thought to function by mediating interactions with ubiquitylated proteins. The chromatin remodeler, SMARCAD1, interacts with KAP1, a transcriptional corepressor. The SMARCAD1-KAP1 interaction is direct and involves the first SMARCAD1 CUE domain (CUE1) and the RBCC domain of KAP1. Here, we present a structural model of the KAP1 RBCC-SMARCAD1 CUE1 complex based on X-ray crystallography. Remarkably, CUE1, a canonical CUE domain, recognizes a cluster of exposed hydrophobic and surrounding charged/amphipathic residues on KAP1, which are presented in the context of a coiled-coil domain, not in a structure resembling ubiquitin. Together, these data suggest that CUE domains may have a wider function than simply recognizing ubiquitin and the ubiquitin-fold.

## Highlights

• A structural model of the KAP1 RBCC-SMARCAD1 CUE1 complex is presented
• SMARCAD1 CUE1, a canonical UBD, has higher affinity for KAP1 RBCC than monoubiquitin
• CUE1 binds an exposed coiled-coil surface on KAP1, not a domain resembling ubiquitin
• KAP1 interaction surface's main feature is a cluster of exposed hydrophobic residues

## Graphical Abstract

**Figure:**
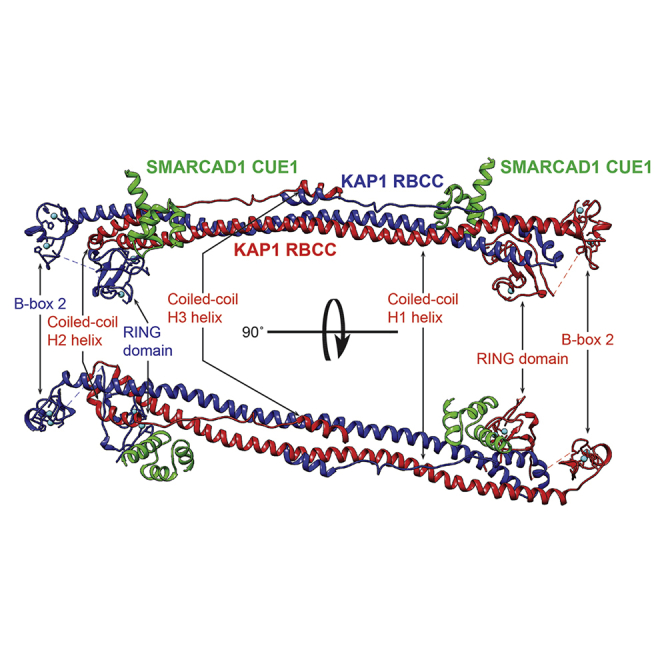

## Teaser

SMARCAD1's CUE1 domain, a canonical CUE domain, is used to allow a tight interaction with the RBCC domain of KAP1 rather than with a domain resembling ubiquitin.

## Introduction

CUE domains are ubiquitin-binding domains (UBDs) that interact with ubiquitin (Ub) by occupying the hydrophobic pocket centered on the highly conserved Ub I44 residue (Hicke et al., 2005, Hofmann, 2009, Husnjak and Dikic, 2012). CUE-Ub interactions are often weak, reflecting a relatively small interaction surface of only ∼400 Å^2^ (Kang et al., 2003, Liu et al., 2012, Prag et al., 2003, Shih et al., 2003). CUE domains have two main conserved sequence elements, a methionine-phenylalanine-proline motif (“MFP”; sometimes “hFP,” or even “haP,” where “h” indicates hydrophobic, and “a” aromatic residues), and a di-leucine repeat (“LL,” sometimes “iL” or even “ih,” where “i” indicates aliphatic, and “h” hydrophobic residues), both of which are essential for Ub binding (Kang et al., 2003, Ponting, 2000, Prag et al., 2003). With the rare exception of Ub-homology domains (UbHs), Ub remains the only known ligand of CUE domains (Hicke et al., 2005, Wilson et al., 2013). As UBDs are essential in detecting the ubiquitylation status of their partner proteins, they are often crucial in mediating the regulation of biological processes by ubiquitylation. Thus, mutation of the UBD of a protein to perturb its interaction with Ub is often a reasonable starting point for interrogating the biological function of that protein.

SMARCAD1 is a candidate for investigation in this manner, as it has a pair of CUE domains and biological functions that merit further mechanistic characterization. SMARCAD1 is a chromatin remodeler, a member of the SWI2/SNF2-like family of enzymes that couple ATP hydrolysis to repositioning, ejecting, or restructuring nucleosomes (Clapier and Cairns, 2009). Functionally, SMARCAD1 has been implicated in facilitating homologous recombination by promoting end-resection, and in maintaining constitutive heterochromatin through DNA replication (Costelloe et al., 2012, Rowbotham et al., 2011).

KAP1 (also known as TRIM28 and TIF1β) is the major interaction partner of SMARCAD1, with the two proteins forming a tight complex (Okazaki et al., 2008, Rowbotham et al., 2011). KAP1 is ubiquitously expressed, and is implicated in transcription repression, heterochromatin formation, and in DNA repair, among other functions (Iyengar and Farnham, 2011).

The CUE domains of SMARCAD1 are, therefore, an intriguing avenue for investigation. Indeed, while our work was being finalized for publication, Mermoud and colleagues reported that mouse SMARCAD1 interacts directly with KAP1 via a CUE1-RBCC interaction in a ubiquitylation-independent manner. Moreover, they demonstrated the functional significance of the CUE1-RBCC interaction in recruiting SMARCAD1 to KAP1 target genes in embryonic stem cells (Ding et al., 2018).

Here, we confirm the direct, ubiquitylation-independent interaction between human SMARCAD1 CUE1 and KAP1 RBCC, before presenting structural and biophysical characterization of this unique interaction.

## Results

### SMARCAD1 and KAP1 Interact Directly in a Ubiquitylation-Independent Manner

**Figure 1:**
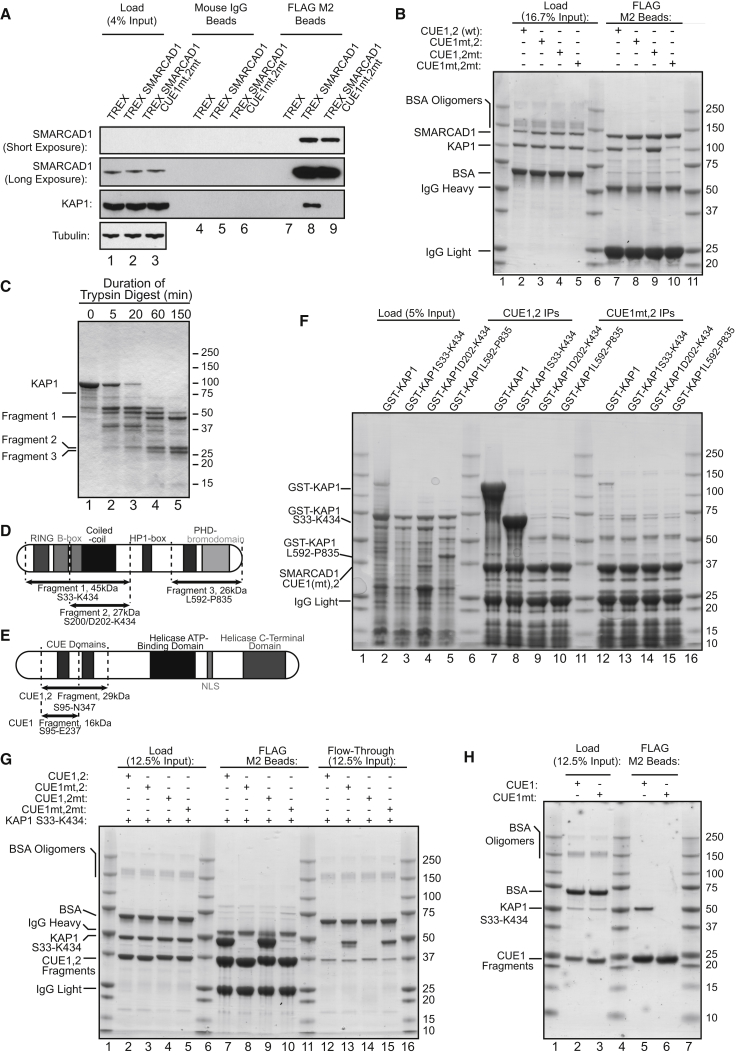
SMARCAD1 CUE1 and KAP1 RBCC Are Necessary and Sufficient for a Direct Interaction (A) Wild-type, but not SMARCAD1 CUE1mt,2mt, coimmunoprecipitates KAP1 (compare lanes 8 and 9) in human cells. (B) The SMARCAD1-KAP1 interaction reconstituted with nonubiquitylated, purified proteins, expressed in *E. coli*. Mutation of SMARCAD1 CUE1 significantly compromises KAP1 binding (compare lanes 7 and 8). (C) Limited tryptic proteolysis of purified recombinant KAP1 yields three main fragments relatively resistant to trypsin. (D) Depiction of the three trypsin-resistant KAP1 fragments, mapped by Edman degradation and intact molecular weight mass spectrometry. (E) Schematic representation of SMARCAD1 CUE1,2 and CUE1 fragments. (F) Immobilized SMARCAD1 CUE1,2 enriches for full-length KAP1 (lane 7) and KAP1 S33-K434 (i.e., fragment 1, lane 8), which spans the RBCC domain, from *E. coli* extract. Binding is specific—mutation of the CUE1 abrogates the interaction (lanes 12 and 13). (G) The SMARCAD1-KAP1 interaction recapitulated *in vitro* with SMARCAD1 CUE1,2 and KAP1 S33-K434 depends on functional CUE1 (compare lane 7 with 8 and 10). (H) SMARCAD1 CUE1 and the KAP1 RBCC are necessary and sufficient (also see Figure S2A) for SMARCAD1-KAP1 interaction.

To study the tandem CUE domains of SMARCAD1 (Figure S1A), we generated human cell lines, depleted of endogenous SMARCAD1 by small hairpin RNA (shRNA) knockdown, which were reconstituted with a doxycycline-inducible, shRNA-resistant gene encoding either FLAG-tagged wild-type SMARCAD1 or SMARCAD1 with point mutations in the CUE domains (“CUE1mt,2mt”) (Figure S1B). The CUE1mt,2mt possesses a total of eight alanine substitutions in the conserved, hydrophobic MFP and LL motifs (FP→AA and LL/LK→AA) that are important for Ub interaction. As expected, KAP1 coimmunoprecipitated with wild-type SMARCAD1 (Figure 1A, lane 8), but CUE domain point mutation abrogated the interaction (lane 9).

We explored whether SMARCAD1 and KAP1 were able to interact directly, independent of ubiquitylation, by testing purified recombinant proteins expressed in *E. coli* in *in vitro* binding assays, exploiting the inability of prokaryotes to ubiquitylate proteins. After mixing, KAP1 coimmunoprecipitated with wild-type SMARCAD1 (Figure 1B, lane 7), while SMARCAD1 CUE1mt,2mt protein showed little or no binding (lane 10), recapitulating the observation in mammalian cells. Notably, point mutation of only the first CUE domain (CUE1mt,2 [FP→AA; LL→AA]) again significantly compromised the ability of SMARCAD1 to bind KAP1 (lane 8), while mutation of the second CUE domain (CUE1,2mt [FP→AA; LK→AA]) had little or no effect (lane 9).

We were also able to reconstitute a stable SMARCAD1-KAP1 complex, with each partner protein in stoichiometric proportions, by sequential affinity purification from a mixture of FLAG-tagged SMARCAD1 and hemagglutinin-tagged KAP1 proteins (Figures S1C and S1D). The reconstituted SMARCAD1-KAP1 complex behaved as a protein complex on gel filtration chromatography (Figure S1E, right panels). We conclude that SMARCAD1 and KAP1 not only interact, but form a highly stable protein complex, in a ubiquitylation-independent manner.

### The RBCC Domain of KAP1 and the First CUE Domain of SMARCAD1 Are Necessary and Sufficient for the SMARCAD1-KAP1 Interaction

The domain architecture of KAP1 is unrevealing regarding the region involved in interacting with the first CUE domain of SMARCAD1 (CUE1). Consequently, limited tryptic digestion was used to identify KAP1 fragments that reflect the tertiary structure of the protein. Three KAP1 fragments, relatively resistant to tryptic digestion (Figure 1C, lane 5), were mapped by Edman sequencing of their N termini and intact molecular weight mass spectrometry (Figure S1F). The largest (“Fragment 1,” S33-K434, 45kDa) spans the RBCC domain, with further cleavage yielding a second fragment, featuring the second B-box and the coiled-coil domain (“Fragment 2,” S200/D202-K434, 27kDa). The final fragment (“Fragment 3,” L592-P835, 26kDa) contains the C-terminal PHD-bromodomain (Figures 1D and S1F).

The fragments were expressed in *E. coli* as GST fusion proteins. Immobilized SMARCAD1 CUE1,2 (S95-N347) (Figure 1E) was then incubated with crude bacterial protein extracts containing these proteins, or full-length GST-KAP1 as a control, to screen these fragments for binding to SMARCAD1. SMARCAD1 CUE1,2 strongly enriched both full-length KAP1 and the S33-K434 RBCC fragment (Figure 1F, lanes 7 and 8). Crucially, this interaction depended on the integrity of SMARCAD1 CUE1: point mutation in this domain completely abrogated binding (lanes 12 and 13).

For confirmation, KAP1 S33-K434 was purified and assessed for binding to four different SMARCAD1 CUE1,2 fragments—the wild-type fragment and those bearing mutations in one or both CUE domain(s). Stoichiometric quantities of KAP1 S33-K434 bound to wild-type CUE1,2 and the CUE1,2mt mutant (Figure 1G, lanes 7 and 9). As expected, KAP1 S33-K434 was incapable of interacting with either the CUE1mt,2 mutant (i.e., inactive CUE1), or the CUE1mt,2mt double mutant (lanes 8 and 10). Moreover, a purified wild-type CUE1 fragment (S95-E237), but not its CUE1mt mutant version, bound purified KAP1 S33-K434 (Figure 1H, compare lanes 5 and 6), confirming that the second CUE domain of SMARCAD1 is dispensable for SMARCAD1-KAP1 interaction. Finally, when the minimal trypsin-resistant fragment, SMARCAD1 N142-R206 (Figure S1G), essentially the CUE1 domain with ∼10–15 flanking amino acids, was coexpressed with the KAP1 RBCC (S33-K434) in *E. coli*, it formed a stable complex that coeluted over at least three columns, including a cation exchange column and gel filtration column (Figure S2A).

Together, these data demonstrate that the first CUE domain of SMARCAD1 and the RBCC domain of KAP1 (S33-K434) are both necessary and sufficient to mediate a direct interaction between SMARCAD1 and KAP1. Ding et al. (2018) reached similar conclusions.

### Crystal Structure of the KAP1 RBCC-SMARCAD1 CUE1,2 Complex

CUE domains normally recognize Ub, so we reasoned that an excess of Ub might be able to interfere with the SMARCAD1-KAP1 interaction. However, even 100 -fold (Figure S2B, lane 10) or a 1,000-fold (data not shown) molar excess of mono-Ub failed to affect binding of SMARCAD1 CUE1,2 to KAP1 S33-K434. We also measured affinity constants for relevant interactions by isothermal titration calorimetry (ITC). Binding of KAP1 RBCC (S33-434) to either SMARCAD1 CUE1 or SMARCAD CUE1,2 was exothermic, with nanomolar dissociation constants (K_d_) of ∼158 and ∼210 nM, respectively. In contrast, mono-Ub bound SMARCAD1 CUE1 and SMARCAD CUE1,2 with high micromolar affinity in an endothermic fashion, with dissociation constants of ∼952 and ∼389 μM, respectively (Figure S2C; Table S1). This is in overall accord with the K_d_ of 1.8 mM determined by NMR for the interaction between mono-Ub and SMARCAD1 CUE1 (West, 2012). Together, these data show that the affinity of the CUE1 domain of SMARCAD1 for the KAP1 RBCC domain is over 1,000-fold greater than that for mono-Ub.

**Figure 2:**
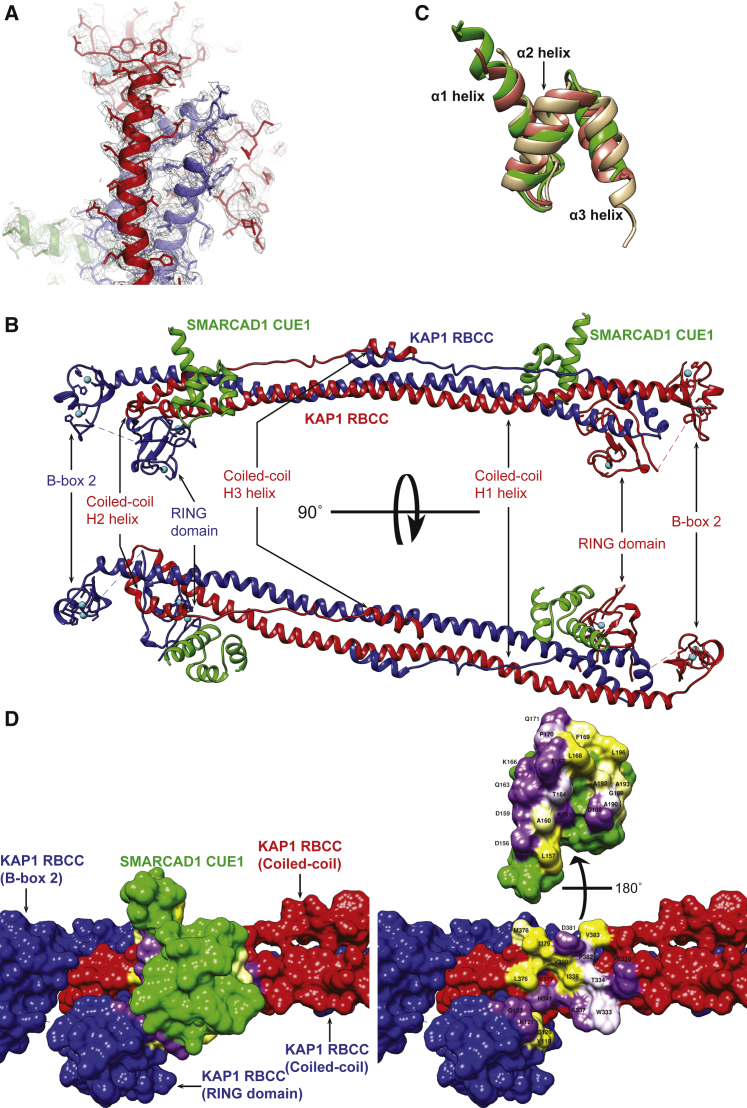
A Structural Model of the KAP1 RBCC ΔBBX1-SMARCAD1 CUE1,2 Complex (A) Electron density map of the KAP1 RBCC ΔBBX1-SMARCAD1 CUE1,2 complex (tetragonal form). One KAP1 RBBC chain is colored red, the other blue, and the CUE1 domain green. (B) KAP1 RBCC homodimerizes in an antiparallel fashion, mediated by the coiled-coil domains. The RING and B-box domains are located at either end of the coiled coil. A SMARCAD1 CUE1 domain binds to each end of the KAP1 RBCC dimer by recognizing an exposed surface of the coiled-coil domain. Domains are colored as in (A). (C) SMARCAD1 CUE1 (green) resembles canonical CUE domains. The CUE domains of CUE2p (tan) and gp78 (salmon) are superimposed. (D) The SMARCAD1 CUE1-KAP1 RBCC interaction surface. On the right, CUE1 domain has been rotated 180° off KAP1. Residues involved in the interaction are labeled and colored by hydrophobicity: yellow, hydrophobic; white, hydroneutral; and purple, hydrophilic.

We now crystallized the KAP1 RBCC domain (G53-K434) in complex with the SMARCAD1 CUE1,2 fragment (L94-N347). The structure was initially determined from a cubic crystal form to 5.5 Å resolution by single-wavelength anomalous diffraction, using the intrinsic anomalous signal of zinc ions (Table S2; Figures S3A and S3B). During model building and refinement, significant disordered sections of KAP1 were noted in the electron density maps. To improve the diffraction resolution, a KAP1 RBCC construct lacking residues 141–202 (KAP1 RBCC ΔBBX1) was produced. Crystals of the KAP1 RBCC ΔBBX1-SMARCAD1 CUE1,2 complex were tetragonal, and diffracted to 3.7 Å resolution (Table S2). The structure was solved by molecular replacement using the cubic crystal structure as a search model. The good quality electron density of the tetragonal crystal form allowed a full atomic model to be built, aided by the ability to determine the sequence register unequivocally at sites of zinc ion coordination and at breaks in secondary structural elements (Figure 2A).

Similar to other TRIM family members (Goldstone et al., 2014, Sanchez et al., 2014, Weinert et al., 2015), the biological unit is a single, elongated dimer (Figure 2B), which is symmetrical in the tetragonal crystal form (KAP1 RBCC ΔBBX1-SMARCAD1 CUE1,2 complex) but slightly asymmetric in the cubic crystal form (KAP1 RBCC-SMARCAD1 CUE 1,2 complex), suggesting that the coiled-coil domains may have a somewhat dynamic structure (Figure S3D). In both cases, the dimer interface is extensive (∼5100 Å^2^) and formed primarily by the antiparallel association of the coiled-coil domains, with some contributions from the RING and second B-box domains. The coiled-coil domain has long and short arms separated by a hairpin turn. The long arm is a continuous, extended helix (H1) spanning ∼170 Å, packed against which is the short arm, comprised of two short helices (H2 and H3) separated by an extended coil (Figure 2B).

Extending from the N-terminal end of each coiled-coil domain, the RING and second B-box domains associate closely with the hairpin turn region of their symmetry mate (Figure 2B). The RING and second B-box domains each have a three-stranded antiparallel β sheet, a short helix and a pair of coordinating zinc ions (Figure S3E). Electron densities for the first B-box domain could not be seen in the cubic form crystals (KAP1 RBCC-SMARCAD1 CUE 1,2 complex) (Figure S3B), nor could its zinc ions be located in anomalous difference maps, suggesting that this domain does not associate with the core, an interpretation supported by limited proteolysis experiments (data not shown).

In both structures, electron density was only observed for a single CUE domain (Figures 2A and S3B), which we conclude must be SMARCAD1 CUE1 based on our biochemical data. The SMARCAD1 CUE1 domain is formed of three helices and is similar in structure to the CUE domains of the yeast CUE2p and human gp78 proteins (Kang et al., 2003, Liu et al., 2012) (root-mean-square deviation = ∼1.1Å) (Figure 2C). Reassuringly, our model of SMARCAD1 CUE1 is comparable with the structure of the small, isolated domain determined independently by NMR (West, 2012).

**Figure 3:**
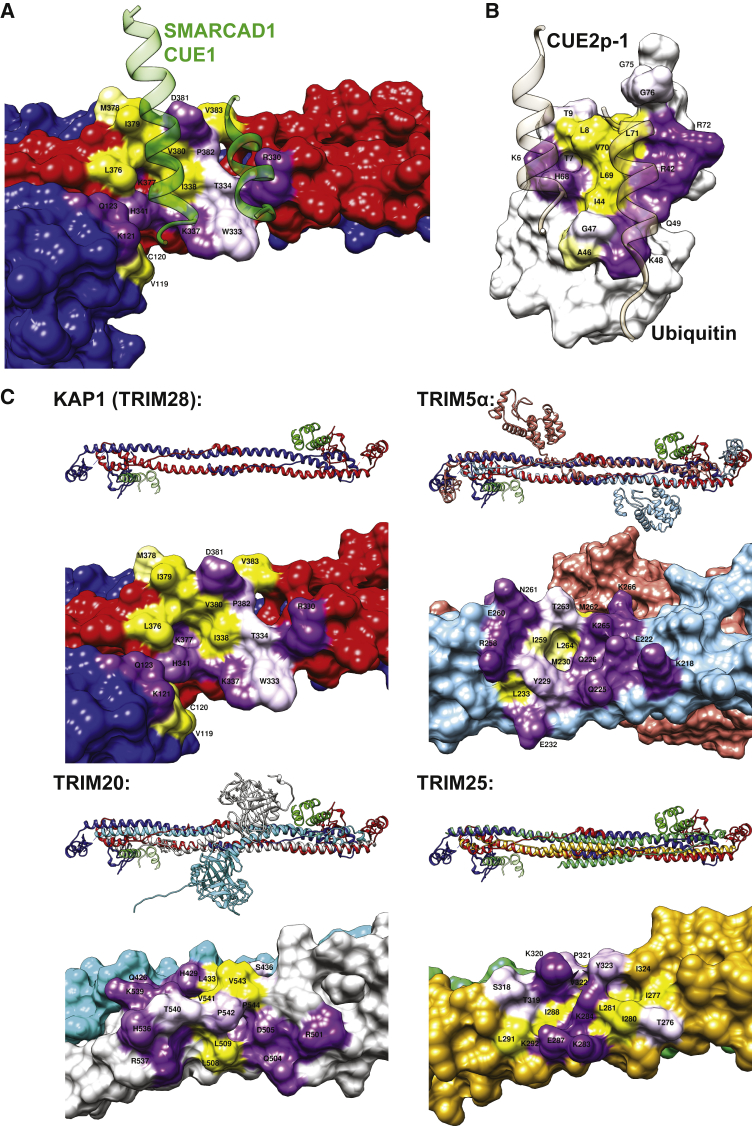
Analysis of the KAP1 RBCC-SMARCAD1 CUE1 Interaction Surface (A) The KAP1 interaction surface features exposed hydrophobic residues (including I338, L376, M378, I379, and V380), surrounded by charged and amphipathic residues. The α1 helix of SMARCAD1 CUE1 (green) overlies and buries the exposed hydrophobic residues on the KAP1 coiled coil. Residues involved in the interaction are colored by hydrophobicity as in Figure 2D. For clarity, the CUE1 α2 helix is not shown. (B) Ub in complex with CUE2p-1 (tan), displayed, orientated and colored as in (A). The CUE domain, via its α1 and α3 helices collectively, fills a hydrophobic pocket (formed by L8, V70, and I44), with surrounding electrostatic interactions. This interaction is distinct from the KAP1 RBCC-SMARCAD1 CUE1 interaction, compare (A) and (B). (C) The KAP1 (TRIM28) RBCC is similar to those of other TRIM proteins, although the precise geometry of the antiparallel coiled coil differs. The pair of exposed hydrophobic clusters on KAP1 is not a conserved structural element, based on hydrophobicity analysis of equivalent surfaces on other TRIM proteins.

Our structural model shows a single SMARCAD1 CUE1 domain bound to each end of the KAP1 coiled-coil dimer. The majority of contacts (i.e., ∼520 Å^2^ of a total interface area of ∼600 Å^2^) are between CUE1 and an exposed surface at the C-terminal end of the long arm of the coiled-coil domain of one KAP1 subunit, but the interface also involves the RING domain of the other KAP1 subunit of the homodimer (Figures 2B and 2D). The interaction surface on the KAP1 coiled-coil domain is formed by a cluster of exposed hydrophobic residues surrounded by charged and amphipathic residues (Figures 2D and 3A). This is matched on the SMARCAD1 CUE1 interaction surface by a corresponding set of hydrophobic residues that are buried in the complex, flanked by charged residues (Figure 2D).

From these data, SMARCAD1 CUE1 and KAP1 RBCC domains appear *prima facie* to resemble other CUE and TRIM RBCC domains respectively. Nevertheless, they succeed in associating directly and specifically with each other. Remarkably, it also provides clear evidence that the SMARCAD1 CUE1 binds a KAP1 domain bearing no structural resemblance to Ub.

### The SMARCAD1 CUE1 α1 Helix Is Principally Responsible for the Specific Interaction with the KAP1 RBCC

Next, we compared the interaction interface of KAP1 RBCC-SMARCAD1 CUE1 with that of a canonical CUE-Ub interaction. Previous structural studies suggest that CUE-Ub interactions rely on a hydrophobic pocket, centered on Ub I44, being filled by the conserved hydrophobic MFP (e.g., M19, F20, and P21 of CUE2p-1) and di-leucine motifs (e.g., L46 and L47 of CUE2p-1) of the CUE domain; electrostatic interactions around the hydrophobic pocket further help stabilize this interaction (Kang et al., 2003) (Figures 3B and S4A). The interaction surface area of CUE-Ub interactions is limited; for example, being ∼360 Å^2^ for the interaction between CUE2p-1 and mono-Ub.

A similar exposed cluster of exposed hydrophobic residues is also present on the interaction surface of KAP1 RBCC; however, it is surrounded by charged and amphipathic residues, resulting in a larger interaction surface area of ∼600 Å^2^. The α1 helix of SMARCAD1 CUE1 overlies and buries the exposed KAP1 RBCC hydrophobic cluster that is comprised of residues I338, L376, M378, I379, and V380 (Figure 3A). Thus, CUE1 α1 helix residues such as Q163, T164, E167, and L168 contact the exposed KAP1 RBCC hydrophobic residues, with T164 appearing to be particularly critical. In addition, the C-terminal end of the CUE1 α1 helix (i.e., K166, P170, Q171) also shares a few contacts with the RING domain of the other subunit of the KAP1 homodimer. As L168, F169, and P170 represent the conserved MFP motif in SMARCAD1 CUE1, KAP1 I338 plays a role most comparable with that of Ub I44 through its interaction with L168. The CUE1 α3 helix—despite containing the conserved di-leucine repeats, L195 and L196—contributes only minimally to the interaction interface via contacts with the peripheral charged/amphipathic KAP1 residues (e.g., R330, W333, and T334) (Figures 2D and 3A).

**Figure 4:**
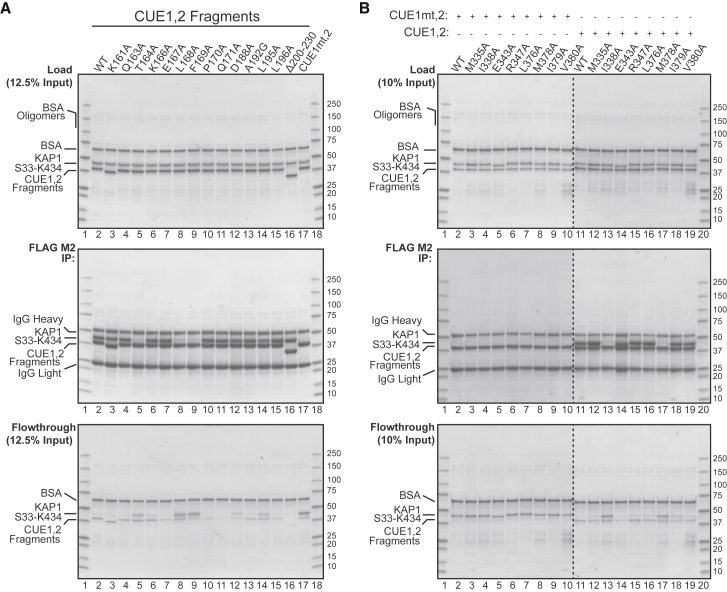
Validation of the SMARCAD1 CUE1,2-KAP1 RBCC Structure by Mutagenesis (A) Effect on the SMARCAD1-KAP1 interaction of amino acid changes in the SMARCAD1 CUE1,2. (B) As in (A), but amino acid substitutions made in KAP1 RBCC (S33-K434).

The exposed surface formed by the SMARCAD1 CUE1 α1-α3 helices used to interact with KAP1 RBCC is, notably, comparable with that employed by other CUE (e.g., CUE2p-1) and UBA domains (e.g., Dsk2 UBA) for canonical binding to mono-Ub and Ub-like (UbL) domains (Figures 3B and S4A). The precise residues employed for Ub recognition vary for each specific UBD, but these surfaces all have exposed hydrophobic residues encircled by hydrophilic or polar residues (Figure S4A). However, while the SMARCAD1 CUE1 α1 helix dominates the interaction with KAP1 RBCC, in canonical Ub binding, the α3 helices of UBDs typically make a greater contribution to filling the hydrophobic pocket of Ub or UbL domains, for example via their conserved di-leucine repeats (Figures 3B and S4A). It is unlikely that SMARCAD1 CUE1 can bind simultaneously to KAP1 RBCC and Ub, as the surface that would recognize Ub canonically is already used and optimized to bind KAP1 RBCC (Figure S4A). Conserved residues, including the MFP motif, retain important roles in the KAP1 RBCC-SMARCAD1 CUE1 interaction, but additional residues, for instance T164 (Figure 4A), have acquired unique, critical roles to enable KAP1 RBCC binding.

### The KAP1 Interaction Surface Is Not Conserved among TRIM Proteins

The previously reported structures of TRIM5α, TRIM20, and TRIM25 (Goldstone et al., 2014, Sanchez et al., 2014, Weinert et al., 2015) were compared with that of KAP1 (Figure 3C). Although all the TRIM proteins have a similar, central antiparallel coiled coil, each coiled coil differs in its precise geometry, with each helix revealing a varying degree of deviation off its axis. Importantly, none of the other TRIM proteins share a similar pattern of exposed residues on their coiled-coil domains in a comparable region to where SMARCAD1 CUE1 binds KAP1, suggesting that this is not a conserved architectural feature of TRIM proteins, but is unique to KAP1 (Figure 3C).

Rittinger and colleagues recently elucidated the crystal structure of the coiled-coil domain of TRIM25 in complex with either the TRIM25 PRYSPRY domain or the influenza A nonstructural protein 1 (NS1) (Koliopoulos et al., 2018). These interactors bind to opposite sides of the exterior surface of the TRIM25 coiled coil, in a region comparable to that on KAP1 recognized by SMARCAD1 CUE. In fact, although the amino acid contacts underpinning these distinct interactions are considerably different, it is striking that the PRYSPRY domain recognizes a surface on the TRIM25 coiled coil that is nearly equivalent to that on KAP1 bound by SMARCAD1 CUE1 (Figure S4B).

### Validation of the Structural Model by Mutagenesis

To validate our structural model, residues identified as being potentially important for the interaction were mutated to alanine in the SMARCAD1 CUE1,2 and KAP1 RBCC fragments. In addition, the four residues cotargeted in the CUE1mt,2 mutant were also individually mutated. Notably, SMARCAD1 T164A, L168A, or F169A mutation abrogated the ability of the CUE1,2 fragment to bind KAP1 RBCC to the same extent as the quadruple CUE1 mutation (Figure 4A, lanes 5, 8, 9, and 17). Our structure indicates that SMARCAD1 T164 forms extensive contacts with the cluster of hydrophobic KAP1 RBCC residues, including I338, I379, V380, and D381. SMARCAD1 L168 and F169 primarily interact with the charged/amphipathic residues (e.g., KAP1 W333, T334, K337) that surround the hydrophobic cluster, but L168 also directly contacts the hydrophobic cluster residue, I338. It is also possible that part of the reason that F169 is indispensable for binding is through a structural role in breaking the α1 helix (Figures 2D, right and 3A).

We next investigated whether mutations targeting these KAP1 residues would also affect SMARCAD1 binding. Gratifyingly, mutation of KAP1 I338 and M378 (Figure 4B, lanes 13 and 17) of the exposed hydrophobic cluster completely abrogated binding. Somewhat curiously, despite the prominent effect of I338A and M378 mutations, we noted that other mutations targeting adjacent residues in the hydrophobic cluster (i.e., L376A, I379A, and V380A) did not perceptibly affect binding (Figure 4B, lanes 16, 18, and 19). Nevertheless, these results together empirically support a mechanism of SMARCAD1 CUE1 binding KAP1 that requires contacts with both the exposed hydrophobic cluster, and the surrounding charged/amphipathic periphery on the coiled-coil domain.

The CUE1mt,2 fragment used in Figure 1 is a composite of the F169A, P170A, L195A, and L196A mutations, and was designed to target both the conserved MFP and LL motifs. Strikingly, the inability of the CUE1mt,2 to bind KAP1 is largely due to the F169A mutation, as neither P170A, L195A, nor L196A point mutation individually had a noticeable effect on binding (Figure 4A, compare lanes 10, 14, and 15 with lane 9 and 17). The absence of an effect from L195A or L196A point mutation is explained from the structure: the α1 helix of SMARCAD1 CUE1 is solely responsible for overlying and burying the exposed KAP1 RBCC hydrophobic cluster, whereas the α3 helix, which contains the di-leucine repeats, makes only a minor contribution to the interaction interface via contacts with peripheral charged/amphipathic KAP1 residues. Indeed, SMARCAD1 CUE1 L195 and L196 are spatially distant from the crucial exposed hydrophobic residues on KAP1 (Figure 3A). Thus, despite its resemblance to canonical CUE domains, these observations reiterate that SMARCAD1 CUE1 is specifically adapted for binding KAP1, in a manner distinct from canonical CUE-Ub interactions, where it would be typically expected for both the MFP motif and di-leucine repeats to contribute directly to occupying the hydrophobic pocket of Ub (Kang et al., 2003).

The SMARCAD1 CUE1 domain, via K166, P170, and Q171 (see Figure 2D, right), also appears to form contacts with the RING domain of the other subunit of the KAP1 homodimer. However, alanine substitution of these residues had no noticeable effect on binding (Figure 4A, lanes 6, 10, and 11). The SMARCAD1 L168A and F169A point mutations had prominent effects in disrupting SMARCAD1-KAP1 binding (Figure 4A, lanes 8 and 9), and would be expected to interfere with contact formation with the charged/amphipathic residues that surround the exposed hydrophobic cluster on KAP1 (Figure 2D). However, other SMARCAD1 mutations (e.g., K161A, E167A, D188A, A192G, and L196A), similarly designed to target interactions with surrounding charged/amphipathic KAP1 residues, all had no discernible effect on the interaction (Figure 4A, lanes 3, 7, 12, 13, and 15). Thus, not all the SMARCAD1 CUE1 interactions with the charged/amphipathic residues surrounding the KAP1 hydrophobic cluster are individually critical for binding, but the possibility that they nevertheless contribute to improving the overall affinity cannot be excluded.

Collectively, mutagenesis empirically validates our structure and supports the theory that the mechanism underpinning the SMARCAD1-KAP1 interaction is recognition of an exposed hydrophobic cluster and surrounding charged/amphipathic residues on the KAP1 coiled coil by SMARCAD1 CUE1.

## Discussion

### CUE Domains Mediate Protein Interactions

CUE domains are generally regarded as protein interfaces for only one ligand, namely Ub (or, much less commonly, UbH domains) (Hicke et al., 2005, Hofmann, 2009, Husnjak and Dikic, 2012). Here, however, we show that the first CUE domain of SMARCAD1—a classical CUE domain—recognizes a ligand structurally distinct from that of Ub, by which it mediates a stable, direct protein-protein interaction with KAP1. Notably, the affinity SMARCAD1 CUE1 displays for KAP1 RBCC (K_d_ ≈ 158 nM) is far higher than that for mono-Ub (K_d_ ≈ 952 μM). Specifically, SMARCAD1 CUE1 recognizes an exposed cluster of hydrophobic residues and surrounding charged/amphipathic residues situated on the exterior surface of the KAP1 coiled-coil domain. Interestingly, the SMARCAD1 CUE1 α1 helix dominates the interaction with KAP1 RBCC and is solely responsible for overlying and burying the exposed hydrophobic cluster, whereas canonical Ub binding typically relies on the combined surface of the α1-α3 helices of UBDs to fill the hydrophobic pocket of Ub. Importantly, it raises the point that when interrogating the function of an uncharacterized CUE domain (or perhaps even UBDs in general), the possibility of it mediating protein-protein interactions beyond interactions with ubiquitylated partner proteins, should also be considered. The relevance *in vivo* is underscored by the finding that mutations in the SMARCAD1 CUE domain disrupt what is normally a very stable SMARCAD1-KAP1 complex. Indeed, while our work was being finalized for publication, Mermoud and colleagues reported the direct interaction between mouse SMARCAD1 and KAP1 via a CUE1-RBCC interaction, in excellent agreement with the conclusions reported here, and further showed that the CUE1-RBCC interaction is functionally important for recruiting SMARCAD1 to KAP1 target genes in stem cells (Ding et al., 2018). It is thus clear that the CUE1-RBCC interaction is of great significance for a stable SMARCAD1-KAP1 complex, not only *in vitro* but also *in vivo*.

Ub-UBD interactions are typically weak, with dissociation constants in the 100-μM range (Kang et al., 2003, Liu et al., 2012, Prag et al., 2003, Shih et al., 2003). One inevitable question raised by our observations is the mechanism by which a CUE interaction can be rendered significantly more stable than classical Ub-UBD binding. Although our model has not provided a conclusive answer, one possibility for the enhanced affinity of the SMARCAD1 CUE1-KAP1 RBCC interaction is the expanded interaction surface area of ∼600 Å^2^ compared, for instance, with ∼360 Å^2^ for the CUE2p-1-mono-Ub interaction (compare Figures 3A and 3B). Another consideration is that the modest dissociation constants for Ub-based interactions are typically measured for the interaction between a UBD and mono- (or di-)Ub in isolation. These values may artificially suggest weaker interactions than reality, since a weak UBD-Ub interaction, assisted by additional specificity domains, could result in an overall stable protein-protein interaction (Hofmann, 2009, Rahighi and Dikic, 2012). Yet, the SMARCAD1-KAP1 interaction is distinctive in being very stable, but apparently dependent only on the CUE1-RBCC interaction, a phenomenon also observed between the proteins when expressed at normal levels in human cells.

It is worth noting that some conserved residues which mediate Ub binding in other CUE domains are also functionally essential for KAP1-binding in SMARCAD1 CUE1. This, combined with the considerable sequence variation among the large family of CUE domains, make it impossible to predict which, if any, among the other CUE domain proteins might also have ligands other than Ub. Overall, our findings nevertheless raise the possibility that, besides functioning as UBDs, CUE domains may potentially fulfill a more general role in mediating protein-protein interactions. Further work will obviously be required to confirm the generalizability of this hypothesis.

### The TRIM RBCC Domain as an Interaction Interface

Our structural model of the SMARCAD1 CUE1-KAP1 RBCC complex confirms that the KAP1 RBCC adopts a structural architecture comparable with other TRIM proteins. It also complements the structure of the C-terminal KAP1 PHD-bromodomains (Ivanov et al., 2007). The elongated, central coiled coil of TRIM proteins is accessorized by N-terminal RING and B-box domains at its apices, but also by additional C-terminal protein domains protruding from its center (Goldstone et al., 2014, Sanchez et al., 2014, Weinert et al., 2015). This modular assembly of proteins domains render TRIM proteins adept as scaffold proteins, recruiting molecular machinery to specific cellular or genomic locations.

Notably, we show that the SMARCAD1-KAP1 interaction does not occur via a discrete UbH domain in KAP1. Rather, the SMARCAD1 CUE1 recognizes an exposed surface created by homodimerization of the KAP1 coiled-coil domains. It was recently reported that the TRIM25 PRYSPRY domain and influenza A NS1 bind to the opposite sides of the TRIM25 coiled-coil domain, although simultaneous binding is impermissible (Koliopoulos et al., 2018). Notably, the surface on TRIM25 recognized by the PRYSPRY domain is comparable with that on KAP1 used for SMARCAD1 CUE binding. It is unclear why the ends of the coiled-coil domains appear to be interaction hotspots, although it is possible that proximity of the flexible linker between the H2 and H3 helices to that region of the coiled coil allows unique interaction surfaces to be created without disrupting the intermolecular packing of the H1 helices. Intriguingly, protein-protein interactions involving the exterior surface of coiled-coil domains may be a general feature of TRIM proteins, although *a priori* predictions of possible protein interactions are difficult given the unique geometry of the helix of each TRIM protein. Despite these caveats, our data support a model of TRIM proteins functioning as an interaction interface by two mechanisms—first, through discrete protein domains that autonomously mediate protein-protein interactions, and second, by supporting interactions that involve exposed surfaces created by oligomerization of the coiled-coil domain.

## STAR★Methods

### Key Resources Table

**Table:** 

REAGENT or RESOURCE	SOURCE	IDENTIFIER
**Antibodies**		

Rabbit polyclonal anti-SMARCAD1	Bethyl	A301-593A; RRID: AB_1078836
Rabbit polyclonal anti-KAP1	Abcam	Ab10483; RRID: AB_297222
Mouse monoclonal anti-α-Tubulin	Francis Crick Institute	TAT-1; RRID: AB_10013740
Mouse monoclonal anti-HA	Francis Crick Institute	12CA5; RRID: AB_2532070
Rabbit polyclonal anti-FLAG	Sigma	F7425; RRID: AB_439687
Sheep anti-mouse IgG HRP-linked F(ab’)_2_ fragments	GE Healthcare	NA9310; RRID: AB_772193
Donkey anti-rabbit IgG HRP-linked F(ab’)_2_ fragments	GE Healthcare	NA9340; RRID: AB_772191

**Bacterial and Virus Strains**		

*E. coli* BL21-CodonPlus® (DE3)-RIL	Agilent	#230245
*E. coli* BL21-CodonPlus® (DE3)-RP	Agilent	#230255
*E. coli* BL21(DE3) Rosetta™ 2	Novagen	71400
*E. coli* BL21(DE3)-R3-pRARE2	Structural Genomics Consortium, Oxford	N/A

**Chemicals, Peptides, and Recombinant Proteins**		

FLAG-SMARCAD1 (WT)	This Study	N/A
FLAG-SMARCAD1 CUE1mt	This Study	N/A
FLAG-SMARCAD1 CUE2mt	This Study	N/A
FLAG-SMARCAD1 CUE1mt,2mt	This Study	N/A
FLAG-CUE1,2 (WT)	This Study	N/A
FLAG-CUE1mt,2	This Study	N/A
FLAG-CUE1,2mt	This Study	N/A
FLAG-CUE1mt,2mt	This Study	N/A
FLAG-CUE1,2 K161A	This Study	N/A
FLAG-CUE1,2 Q163A	This Study	N/A
FLAG-CUE1,2 T164A	This Study	N/A
FLAG-CUE1,2 K166A	This Study	N/A
FLAG-CUE1,2 E167A	This Study	N/A
FLAG-CUE1,2 L168A	This Study	N/A
FLAG-CUE1,2 F169A	This Study	N/A
FLAG-CUE1,2 P170A	This Study	N/A
FLAG-CUE1,2 Q171A	This Study	N/A
FLAG-CUE1,2 D188A	This Study	N/A
FLAG-CUE1,2 A192G	This Study	N/A
FLAG-CUE1,2 L195A	This Study	N/A
FLAG-CUE1,2 L196A	This Study	N/A
FLAG-CUE1,2 Δ200-230	This Study	N/A
FLAG-CUE1	This Study	N/A
FLAG-CUE1mt	This Study	N/A
HA-KAP1	This Study	N/A
KAP1 G53-K434	This Study	N/A
KAP1 G53-K434 ΔBBX1 (Δ141-202)	This Study	N/A
KAP1 S33-K434	This Study	N/A
KAP1 S33-K434 M335A	This Study	N/A
KAP1 S33-K434 I338A	This Study	N/A
KAP1 S33-K434 E342A	This Study	N/A
KAP1 S33-K434 R347A	This Study	N/A
KAP1 S33-K434 L376A	This Study	N/A
KAP1 S33-K434 M378A	This Study	N/A
KAP1 S33-K434 I379A	This Study	N/A
KAP1 S33-K434 V380A	This Study	N/A
Ubiquitin	Boston Biochem	U-100H
Ulp1	Purified as per Peter Cherepanov, FCI	N/A
Lysozyme	Sigma	L6876
Micrococcal nuclease	NEB	M0247
Ni-NTA agarose	Qiagen	30250
HisTrap HP	GE Healthcare	17524801
HiTrap Heparin HP column	GE Healthcare	17040701
ProSwift WCX-1S	Thermo Fisher Scientific	064295
ProSwift SAX-1S	Thermo Fisher Scientific	064293
MAbPac SEC-1 column	Thermo Fisher Scientific	074696
Mono Q™ 5/50 GL column	GE Healthcare	17-5166-01
HiPrep 16/60 Sephacryl S-400 HR	GE Healthcare	28-9356-04
Mono Q™ 10/100 GL	GE Healthcare	17-5167-01
HiLoad 16/600 Superdex 200 column	GE Healthcare	28-9893-35
Amicon Ultra-15 30K MWCO spin concentrator	Millipore	UFC903008
Amicon Ultra-4 10K MWCO spin concentrators	Millipore	UFC801024
Microcon-30kDa Centrifugal Filter Unit	Millipore	MRCF0R030
anti-HA (3F10) affinity matrix	Roche	11 815 016 001; RRID: AB_390914
HA peptide	Peptide Chemistry STP, FCI	N/A
anti-FLAG M2 affinity gel	Sigma	A2220; RRID: AB_10063035
FLAG peptide	Peptide Chemistry STP, FCI	N/A
Bio-Rad Protein Assay	Bio-Rad	5000006
InstantBlue™	Expedeon	ISB1L
SilverQuest Silver Staining Kit	Invitrogen	LC6070
SYPRO® Ruby	Thermo Fisher Scientific	S11791

**Deposited Data**		

TRIM25 coiled-coil domain X-ray structure	PDB	PDB 4CFG
TRIM28 B-box 2 domain X-ray structure	PDB	PDB 2YVR
TRIM56 RING domain X-ray structure	PDB (Fiskin et al., 2017)	PDB 5JW7
GP78 CUE domain NMR structure	PDB (Liu et al., 2012)	PDB 2LVN
KAP1 RBCC-SMARCAD1 CUE1,2 X-ray structure	This Study	PDB 6H3A
KAP1 RBCC ΔBBX1-SMARCAD1 CUE1,2 X-ray structure	This Study	PDB 6QU1

**Experimental Models: Cell Lines**		

T-Rex-293 cell line	Thermo Fisher Scientific	R71007
293 T-Rex F-SMARCAD1 716R	This Study	N/A

**Experimental Models: Organisms/Strains**		

SMARCAD1 cDNA	Human	UniProt Q9H4L7
KAP1 cDNA	Human	UniProt Q13263

**Recombinant DNA**		

pcDNA4/TO vector	Thermo Fisher Scientific	V102020
pSMARCAD1-716R-CUE1mt,2mt/TO	This Study	N/A
pGIPZ-SMARCAD1 V2LHS-51716	Dharmacon	V2LHS-51716
pGEX-6P-1	GE Healthcare	28-9546-48
pGEX6P1-KAP1	This Study	N/A
pGEX6P1-KAP1 S33-K434	This Study	N/A
pGEX6P1-KAP1 D202-K434	This Study	N/A
pGEX6P1-KAP1 L592-P835	This Study	N/A
pET28a-SUMO	Peter Cherepanov, FCI	N/A
pET28a-SUMO-HA-KAP1	This Study	N/A
pET28a-SUMO-KAP1 S33-K434	This Study	N/A
pET28a-SUMO-KAP1 S33-K434 M335A	GenScript, This Study	N/A
pET28a-SUMO-KAP1 S33-K434 I338A	GenScript, This Study	N/A
pET28a-SUMO-KAP1 S33-K434 E342A	GenScript, This Study	N/A
pET28a-SUMO-KAP1 S33-K434 R347A	GenScript, This Study	N/A
pET28a-SUMO-KAP1 S33-K434 L376A	GenScript, This Study	N/A
pET28a-SUMO-KAP1 S33-K434 M378A	GenScript, This Study	N/A
pET28a-SUMO-KAP1 S33-K434 I379A	GenScript, This Study	N/A
pET28a-SUMO-KAP1 S33-K434 V380A	GenScript, This Study	N/A
pET28a-SUMO-F-SMARCAD1	This Study	N/A
pET28a-SUMO-F-SMARCAD1-CUE1mt,2	This Study	N/A
pET28a-SUMO-F-SMARCAD1-CUE1,2mt	This Study	N/A
pET28a-SUMO-F-SMARCAD1-CUE1mt,2mt	This Study	N/A
pET28a-SUMO-F-CUE1,2 (S95-N347)	This Study	N/A
pET28a-SUMO-F-CUE1mt,2	This Study	N/A
pET28a-SUMO-F-CUE1,2mt	This Study	N/A
pET28a-SUMO-F-CUE1mt,2mt	This Study	N/A
pET28a-SUMO-F-CUE1,2 K161A	GenScript, This Study	N/A
pET28a-SUMO-F-CUE1,2 Q163A	GenScript, This Study	N/A
pET28a-SUMO-F-CUE1,2 T164A	GenScript, This Study	N/A
pET28a-SUMO-F-CUE1,2 K166A	GenScript, This Study	N/A
pET28a-SUMO-F-CUE1,2 E167A	GenScript, This Study	N/A
pET28a-SUMO-F-CUE1,2 L168A	GenScript, This Study	N/A
pET28a-SUMO-F-CUE1,2 F169A	GenScript, This Study	N/A
pET28a-SUMO-F-CUE1,2 P170A	GenScript, This Study	N/A
pET28a-SUMO-F-CUE1,2 Q171A	GenScript, This Study	N/A
pET28a-SUMO-F-CUE1,2 D188A	GenScript, This Study	N/A
pET28a-SUMO-F-CUE1,2 A192G	GenScript, This Study	N/A
pET28a-SUMO-F-CUE1,2 L195A	GenScript, This Study	N/A
pET28a-SUMO-F-CUE1,2 L196A	GenScript, This Study	N/A
pET28a-SUMO-F-CUE1,2 Δ200-230	GenScript, This Study	N/A
pET28a-SUMO-CUE1,2	This Study	N/A
pET28a-SUMO-F-CUE1 (S95-E237)	This Study	N/A
pET28a-SUMO-F-CUE1mt	This Study	N/A
pCDF-Duet1-SUMO	Peter Cherepanov, FCI	N/A
pCDF-SUMO-KAP1 S33-K434	This Study	N/A

**Software and Algorithms**		

Origin	Malvern	N/A
DIALS 1.11	DIALS	N/A
Phenix autosol	Phenix	N/A
Phenix refine	Phenix	N/A
PHASER	CCP4	N/A
UCSF Chimera	UCSF	N/A

**Other**		

Diamond Light Source beamline i03	N/A	N/A
Diamond Light Source beamline I04-1	N/A	N/A

### Contact for Reagent and Resource Sharing

Further information and requests for resources and reagents should be directed to and will be fulfilled by the Lead Contact, Jesper Svejstrup (Jesper.Svejstrup@crick.ac.uk).

### Experimental Model and Subject Details

Human 293 T-Rex (Thermo Fisher Scientific) were cultured according to the manufacturer’s instructions in a humidified incubator at 37°C with 5% CO_2_.

Human SMARCAD1 and KAP1 cDNA was used as the template for expression in *E. coli*. The expression vectors used in this study were all sequenced, and the wild type cDNA sequences were confirmed to encode for an amino acid sequence that corresponds exactly with the UniProt reference sequences Q9H4L7 and Q13263 respectively.

### Method Details

#### Generation of Stable Cell Lines

293 T-Rex cells were depleted of endogenous SMARCAD1 by GIPZ lentiviral shRNA (Dharmacon) knockdown, before being rescued with doxycycline-inducible expression of exogenous, shRNA-resistant, FLAG-tagged SMARCAD1 or SMARCAD1 CUE1mt,2mt, using the T-Rex system (ThermoFisher Scientific). Individual colonies were isolated. Doxycycline titration identified concentrations that resulted in exogenous SMARCAD1 being expressed at approximately endogenous levels.

#### Preparation of Cell Extracts & Protein Detection

Soluble bacterial extracts were prepared in GST-L-Zn buffer (20mM Tris, 100mM NaCl, 10% (v/v) glycerol, 0.1% (v/v) NP-40, 50μM ZnSO_4_, 5mM β-ME; pH7.90 at 4°C), treated with lysozyme (2mg/mL, Sigma), sonicated in a Bioruptor (Diagenode), and digested with micrococcal nuclease (2000 gel units/mL, NEB). Mammalian whole cell extracts were prepared in Triton lysis buffer (50mM Tris, 150mM NaCl, 1mM EDTA, 1% (v/v) Triton X-100; pH7.50 at RT) supplemented with 1X protease inhibitor cocktail (284ng/mL leupeptin, 1.37μg/mL pepstatin A, 170μg/mL PMSF, 330μg/mL benzamidine, and sonicated in a Bioruptor® (Diagenode). Protein concentrations were determined using the Bradford assay (Bio-Rad Protein Assay) calibrated with a BSA standard curve.

Criterion™ pre-cast XT Bis-Tris 4-12% or TGX™ 5-15% gradient gels (Bio-Rad) were used for SDS-PAGE. Purified proteins were detected by InstantBlue™ (Expedion), silver (SilverQuest Silver Staining Kit, Invitrogen), or SYPRO® Ruby (ThermoFisher Scientific) staining. Alternatively, Western blotting was performed according to standard techniques using Amersham™ Protran Premium 0.45μm nitrocellulose (GE Healthcare). Membranes were pre-stained with Ponceau S solution (Sigma). The primary antibodies used here were: anti-SMARCAD1 (Bethyl A301-593A) 1:1000, anti-KAP1 (Abcam ab10483) 1:1000, anti-α-tubulin (clone TAT-1) 1:10000, anti-HA (clone 12CA5) 1:10000, and anti-FLAG (Sigma F7425) 1:1000. Either sheep anti-mouse IgG or donkey anti-rabbit IgG HRP-linked F(ab’)_2_ fragments (GE Healthcare) diluted 1:10000 was used as the secondary antibody.

#### Immunoprecipitation

FLAG-tagged proteins were immunoprecipitated using 15μL of anti-FLAG® M2 affinity gel (Sigma) (or mouse IgG beads (Sigma) as controls) from cell extract containing 2.5mg of total protein per reaction. After incubation at 4°C for 3 hours, beads were washed thrice in lysis buffer, and eluted by boiling in SDS loading buffer.

#### Expression & Purification of Recombinant Proteins

SMARCAD1 and its derivatives (e.g. CUE1,2 and CUE1 fragments) were expressed in BL-21 CodonPlus (DE3)-RIL (Stratagene) *E. coli* cells, KAP1 and its derivatives in BL-21 CodonPlus (DE3)-RP (Stratagene), and the SMARCAD1 CUE1,2-KAP1 RBCC complex was co-expressed in Rosetta2 (DE3) cells (Novagen). Expression was induced with 0.5mM IPTG at either 16°C (full-length SMARCAD1) or 30°C (all other constructs), for either 3 (CUE1,2 fragments) or 6 hours (all other constructs).

SMARCAD1 was nickel-affinity purified with a 5mL HisTrap HP column (GE Healthcare), then dialysed overnight at 4°C against P-100 buffer (10mM sodium phosphate, 100mM NaCl, 10%(v/v) glycerol, 5mM β-ME; pH7.50 at 4°C) in the presence of 100μg of recombinant Ulp1 (a SUMO protease). Subsequent chromatographic steps were a 5mL HiTrap Heparin HP column (GE Healthcare), ProSwift WCX-1S (ThermoFisher Scientific) for SMARCAD1 CUE1,2mt and SMARCAD1 CUE1mt,2mt, and then finally ProSwift SAX-1S for all constructs (ThermoFisher Scientific). The final fractions were concentrated using a Microcon spin concentrator (Millipore) with a 50K MWCO and exchanged into P-100 buffer.

SMARCAD1 CUE1,2 and CUE1 fragments were first affinity purified using 3mL of Ni-NTA agarose (Qiagen). The SUMO tag was cleaved by recombinant Ulp1 (140μg) during dialysis against Q-100 buffer (10mM Tris, 100mM NaCl, 10%(v/v) glycerol, 5mM β-ME; pH7.90 at 4°C), and depleted by reloading the sample over the 3mL of Ni-NTA resin and collecting the unbound flow-through. If required, these purifications were followed by ion exchange chromatography on a 1mL Mono Q 5/50 GL column (GE Healthcare). The samples were concentrated with Amicon Ultra-4 10K MWCO spin concentrators (Millipore).

As KAP1 and the KAP1 RBCC contain zinc-finger domains, they were expressed whilst cultured in LB supplemented with 50μM ZnSO_4_ or ZnCl_2_, and all buffers used in the purification protocol contained 50μM ZnSO_4_ or ZnCl_2_. KAP1 was purified using a 5mL HisTrap HP column, dialysed against P-100 buffer in the presence of recombinant Ulp1 (100μg), then loaded onto a 5mL HiTrap Heparin HP column. The eluate was concentrated to a volume of approximately 4mL using an Amicon Ultra-15 30K MWCO spin concentrator, before being loaded onto a 120mL HiPrep 16/60 Sephacryl S-400 HR gel filtration column (GE Healthcare). The sample was re-concentrated using a spin concentrator; the final buffer was GF-150Zn buffer (10mM Tris, 150mM NaCl, 50μM ZnSO_4_, 2mM DTT; pH7.90 at 4°C).

KAP1 RBCC (S33-K434) was purified using 3mL of Ni-NTA agarose, cleaved with recombinant Ulp1 (140μg) during dialysis against Q-100 buffer, and depleted of its SUMO tag as described above. This was followed by chromatography on a 1mL Mono Q 5/50 GL column, and peak fractions dialyzed against Q-100 buffer.

#### Limited Tryptic Proteolysis

Limited tryptic digestion was performed in trypsin buffer (20mM Tris pH7.40, 50mM NaCl, 1mM CaCl_2_, 2mM DTT) using 1/1000 (w/w) the amount of trypsin as purified protein. The reactions were stopped by addition of a protease inhibitor cocktail. For Edman degradation, digested samples were resolved by SDS-PAGE, transferred onto an Amersham Hybond P 0.45 PVDF membrane (GE Healthcare), and stained with Ponceau S (Sigma), following which, selected bands were excised. Edman degradation (5 cycles each) was performed by AltaBioscience.

For intact molecular weight mass spectrometry, the digested samples were first incubated with 50mM DTT to remove β-ME adducts (from the purification buffers). Tryptic peptides were removed using an Ultrafree-CL centrifugal filter unit with a 5K MWCO (Millipore). LC/MS grade formic acid (Fisher Scientific) was added for a concentration of at least 0.2% (v/v) and pH.

#### Reconstitution of SMARCAD1-KAP1 Complex & Analytical Gel Filtration

180μg of purified FLAG-SMARCAD1 was mixed with 180μg of purified HA-KAP1 in SK reconstitution buffer (10mM sodium phosphate, 200mM NaCl, 10% (v/v) glycerol, 0.1% (v/v) NP-40; pH7.50 at RT) and 5mM β-ME. The complex was then reconstituted by sequential affinity purification with anti-HA (3F10) affinity matrix (Roche) and anti-FLAG M2 affinity gel (Sigma). Bound proteins were eluted respectively with HA (1mg/mL) and FLAG peptide (500μg/mL, Peptide Chemistry core facility, Francis Crick Institute), prepared in P-100 buffer.

Analytical gel filtration chromatography was performed using a 4×300mm MAbPac SEC-1 column (ThermoFisher Scientific). 375ng of protein was loaded per run, and eluted isocratically in P-200 GF buffer (10mM sodium phosphate, 200mM NaCl, 2mM DTT; pH7.50 at 4°C).

#### SMARCAD1-KAP1 Binding Assays

For the binding assay with purified full-length SMARCAD1 and KAP1, 7μg of each was mixed together in a 280μL binding reaction containing SK binding buffer (10mM Tris pH7.50 at RT, 150mM NaCl, 10% (v/v) glycerol, 0.01% (v/v) NP-40, 50μM ZnSO_4_), 0.1mg/mL BSA, and 5mM β-ME. The reactions were adjusted to a final sodium chloride concentration of 200mM. The binding reactions were incubated at 4°C for 1 hour before being immunoprecipitated overnight at 4°C with 20μL of anti-FLAG M2 affinity gel (Sigma) per reaction. The beads were washed in 500μL of SK-200 buffer (10mM Tris pH7.50 at RT, 200mM NaCl, 10% (v/v) glycerol, 0.01% (v/v) NP-40, 50μM ZnSO_4_, 5mM β-ME) three times before the beads were eluted with 30μL of 2X SDS-PAGE loading buffer by heating the samples to 100°C for 5 minutes.

The binding assays involving purified fragments of SMARCAD1 (i.e. CUE1,2 and CUE1) and KAP1 (i.e. RBCC) were performed similarly, with the following slight adjustments: 9.6μg of each protein was used in a 240μL binding reaction incubated for 1 hour at 4°C, and immunoprecipitated with 15μL of anti-FLAG M2 affinity gel for 3 hours at 4°C before elution as described above. The effect of ubiquitin on the SMARCAD1-KAP1 interaction was investigated by adding purified recombinant, monomeric ubiquitin (Boston Biochem) to the binding reaction.

The affinity resin of immobilized SMARCAD1 CUE1,2 fragment was prepared by saturating the binding capacity of the M2 resin with three-fold as much purified FLAG-tagged protein (approximately 12.2nmol protein/mL resin), incubating the beads at 4°C overnight, before washing off unbound protein. To 20μL of CUE1,2-coupled resin, *E. coli* extracts containing GST-tagged KAP1 fragments (2.5mg of total protein per reaction) were added and incubated at 4°C for 3 hours. The beads were washed thrice in SK-200 buffer thrice, before being eluted as above.

#### Isothermal Titration Calorimetry

ITC measurements were performed on a MicroCal ITC200 calorimeter (Malvern). All samples were dialysed into buffer containing 10 mM Tris-HCl pH 7.9 at 4°C, 100 mM NaCl and 5 mM β-ME. Titrations of KAP1 RBCC (S33-K434) with SMARCAD1 CUE1 and CUE1,2 were performed at 20°C with KAP1 RBCC (typically 9-18 μM) in the cell and SMARCAD1 CUE1 or CUE1,2 (typically 90-300 μM) in the syringe. Titrations of SMARCAD1 CUE1 or CUE1,2 with mono-Ub were performed at 10°C with 120 μM SMARCAD1 CUE1 or CUE1,2 in the cell and 9.8 mM mono-Ub in the syringe. Due to the low binding affinity between SMARCAD1 CUE1 or CUE1,2 and mono-Ub, a high mono-Ub concentration (9.8 mM) in the syringe was required to measure the interaction. As a result, a highly intense heat of dilution was observed when mono-Ub was titrated into buffer only. To minimise the intensity of the heat of dilution, titrations of SMARCAD1 CUE1 or CUE1,2 with mono-Ub were recorded at 10°C, rather than at 20°C.

Data were analysed using the Origin software supplied by the manufacturer (Malvern) using nonlinear regression with the One set of sites model. For each experiment, the heat associated with ligand dilution was measured and subtracted from the raw data.

#### Crystallization, Structure Determination & Refinement

For crystallization an N-terminally SUMO-tagged KAP1 G53-K434 fragment was overexpressed in *E.coli* BL21(DE3)-R3-pRARE2 cells. Cells were grown at 37°C in TB medium supplemented with 50μg/mL kanamycin until an optical density of 2-3, then induced with 0.3 mM IPTG and incubated overnight at 18°C. Purification was as described above for KAP1 S33-K434, with the exception of the final purification step of size exclusion chromatography using a HiLoad 16/60 Superdex S200 column, where a buffer containing 50mM Hepes pH7.5, 500mM NaCl, 5% glycerol and 0.5mM Tris (2-carboxyethyl) phosphine (TCEP) was used instead. The KAP1 RBCC ΔBBX1 construct was generated from the KAP1 G53-K434 construct by site directed mutagenesis, and was overexpressed and purified as above. Both KAP1 RBCC constructs were concentrated to 10mg/ml using a Millipore 30,000 MWCO centrifugal concentrator and mixed with SMARCAD CUE1,2 (purified as described above) in a 1:1.1 ratio (slight excess of SMARCAD CUE1,2). Crystallization was performed by sitting drop vapour diffusion and crystals of the KAP1 RBCC-SMARCAD1 CUE1,2 complex were grown from conditions containing 1.2M sodium malonate, 0.5% Jeffamine ED-2003 and 0.1M HEPES pH7.0, with a 1:2 protein to precipitant drop ratio. Crystals of the KAP1 RBCC ΔBBX1-SMARCAD CUE1,2 complex were grown from conditions containing 25 % PEG 3350 and 0.1 M HEPES pH 7.5. Crystals were loop mounted and transferred to a cryoprotectant solution comprising the well solution supplemented with 25% ethylene glycol, before being flash-cooled in liquid nitrogen.

A SAD dataset extending to 5.5Å was collected at Diamond Light Source beamline i03 and the data were processed using DIALS (Winter et al., 2018). The structure was solved using Phenix autosol (Terwilliger et al., 2009) using the intrinsic anomalous signal of the zinc ions. The initial phases were improved substantially by solvent flattening, given the extremely high solvent content of 92%, which reflects an unusual crystal packing arrangement – the unit cell of 300Å diameter, is a large proteinaceous cage with internal voids (Figure S3A). Model building was performed using either existing crystal structures of fragments or template derived models, which were directly fitted in to the experimentally phased maps based on zinc ions (i.e. RING and B-box domains) or recognizable secondary structure elements (i.e. coiled-coil and CUE1 domain). Structures used for model building were: TRIM25 coiled-coil domain (PDB 4CFG), TRIM28 B-box 2 domain (PDB 2YVR), TRIM56 RING domain (PDB 5JW7) (Fiskin et al., 2017) and gp78 CUE domain (PDB 2LVN) (Liu et al., 2012). Data were collected on the KAP1 RBCC ΔBBX1-SMARCAD1 CUE 1,2 complex crystals at Diamond Light Source beamline I04-1 and the structure was solved by molecular replacement using the programme PHASER (McCoy et al., 2007) and the structure of the KAP1 RBCC-SMARCAD1 CUE 1,2 complex as a search model. Both structures were refined using Phenix refine (Adams et al., 2010). A summary of the data collection and refinement statistics is shown in Table S1.

Refined structures were visualized and analysed using UCSF Chimera (Pettersen et al., 2004). For comparative analysis, atomic coordinates were obtained from the PDB using accessions PDB: 1OTR (ubiquitin-CUE2-1 complex; Kang et al., 2003), PDB: 1WR1 (Dsk2 UBA-ubiquitin complex; Ohno et al., 2005), PDB: 2BWE (Dsk2 UBA-Dsk2 UBL complex; Lowe et al., 2006), PDB: 4TN3 (TRIM5α; Goldstone et al., 2014), PDB: 4CG4 (TRIM20; Weinert et al., 2015), PDB: 4LTB (TRIM25; Sanchez et al., 2014), PDB: 6FLN (TRIM25 coiled-coil-TRIM25 PRYSPRY complex; Koliopoulos et al., 2018) and PDB: 5NT2 (TRIM25 coiled-coil-NS1 complex; Koliopoulos et al., 2018).

### Quantification and Statistical Analysis

Experiments were repeated at least three times, and representative images presented as figures here.

### Data and Software Availability

The accession numbers for the atomic coordinates and structure factors for the X-ray structures of the cubic form KAP1 RBCC-SMARCAD1 CUE1,2 complex and tetragonal form KAP1 RBCCΔBBX1-SMARCAD1 CUE1,2 complex reported in this paper are PDB: 6H3A and PDB: 6QU1 respectively. Other data and constructs used in this study are available from the corresponding author upon reasonable request.

## Supplemental Information

Document S1. Figures S1–S4 and Tables S1 and S2

Document S2. Article plus Supplemental Information
