# Supplementary material for: A Ubiquitin-Binding Domain that Binds a Structural Fold Distinct from that of Ubiquitin
Source: Structure. 2019 Aug 6;27(8):1316–1325.e6. doi: 10.1016/j.str.2019.05.003 (PMC6688830; doi:10.1016/j.str.2019.05.003)
Supplement: Document S1. Figures S1–S4 and Tables S1 and S2 [file mmc1.pdf]

Structure, Volume *27*

Supplemental Information

A Ubiquitin-Binding Domain that Binds a Structural Fold Distinct from that of Ubiquitin

Michael Lim, Joseph A. Newman, Hannah L. Williams, Laura Masino, Hazel Aitkenhead, Angeline E. Gravard, Opher Gileadi, and Jesper Q. Svejstrup

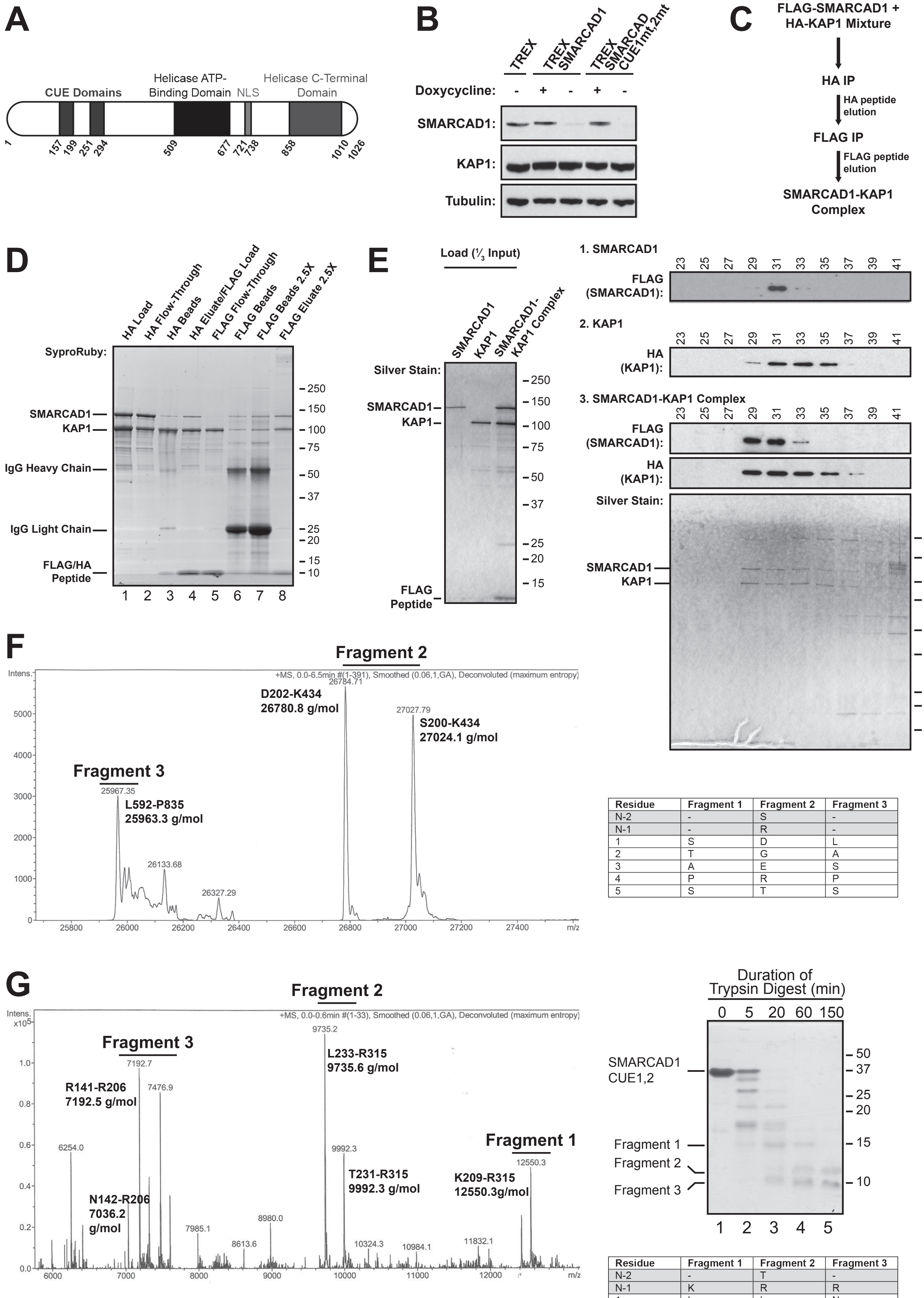

Residue	Fragment 1	Fragment 2	Fragment 3
N-2	-	S	-
N-1	-	R	-
1	S	D	L
2	T	G	A
3	A	E	S
4	P	R	P
5	S	T	S

Residue	Fragment 1	Fragment 2	Fragment 3
N-2	-	T	-
N-1	K	R	R
1	L	L	N
2	S	D	D
3	S	H	D
4	S	G	I
5	-	E	S

Figure S1, related to Figure 1

Figure S1, related to Figure 1. Further characterization of the SMARCAD1-KAP1 interaction in cell lines, by *in vitro* reconstitution, and limited tryptic digestion. A. Domain architecture of the human SMARCAD1 protein, with amino acid positions below. **B.** Stable HEK293 T-REx cell lines, depleted of endogenous SMARCAD1, but inducibly (i.e. + doxycycline) expressing exogenous FLAG-tagged SMARCAD1 at approximately normal levels. The cells were reconstituted with either wild type protein, or SMARCAD1 CUE1mt,2mt. **C.** Sequential affinity purifications performed to reconstitute the SMARCAD1-KAP1 complex *in vitro*. **D.** Representative gel of the SMARCAD1-KAP1 complex reconstitution protocol. The final reconstituted SMARCAD1-KAP1 complex is pure and comprises near-stoichiometric quantities of each protein. **E.** SMARCAD1, KAP1 and the reconstituted SMARCAD1-KAP1 complex (left panels show the samples loaded) assessed by analytical gel filtration chromatography (right panels) confirm that SMARCAD1 and KAP1 of the reconstituted complex (3) precisely co-elute in earlier eluting fractions than SMARCAD1 (1) or KAP1 (2) individually. **F.** Trypsin-resistant KAP1 fragments (see Figure 1C) were mapped by Edman degradation (table) and intact molecular weight mass spectrometry (graph), with good concordance between measured and calculated molecular weights. A peak corresponding to Fragment 1 was not identified by mass spectrometry, but its N-terminus was unequivocally identified by Edman degradation. **G.** Limited tryptic proteolysis of SMARCAD1 CUE1,2 was performed to identify the minimal CUE1 and CUE2 domains as above.

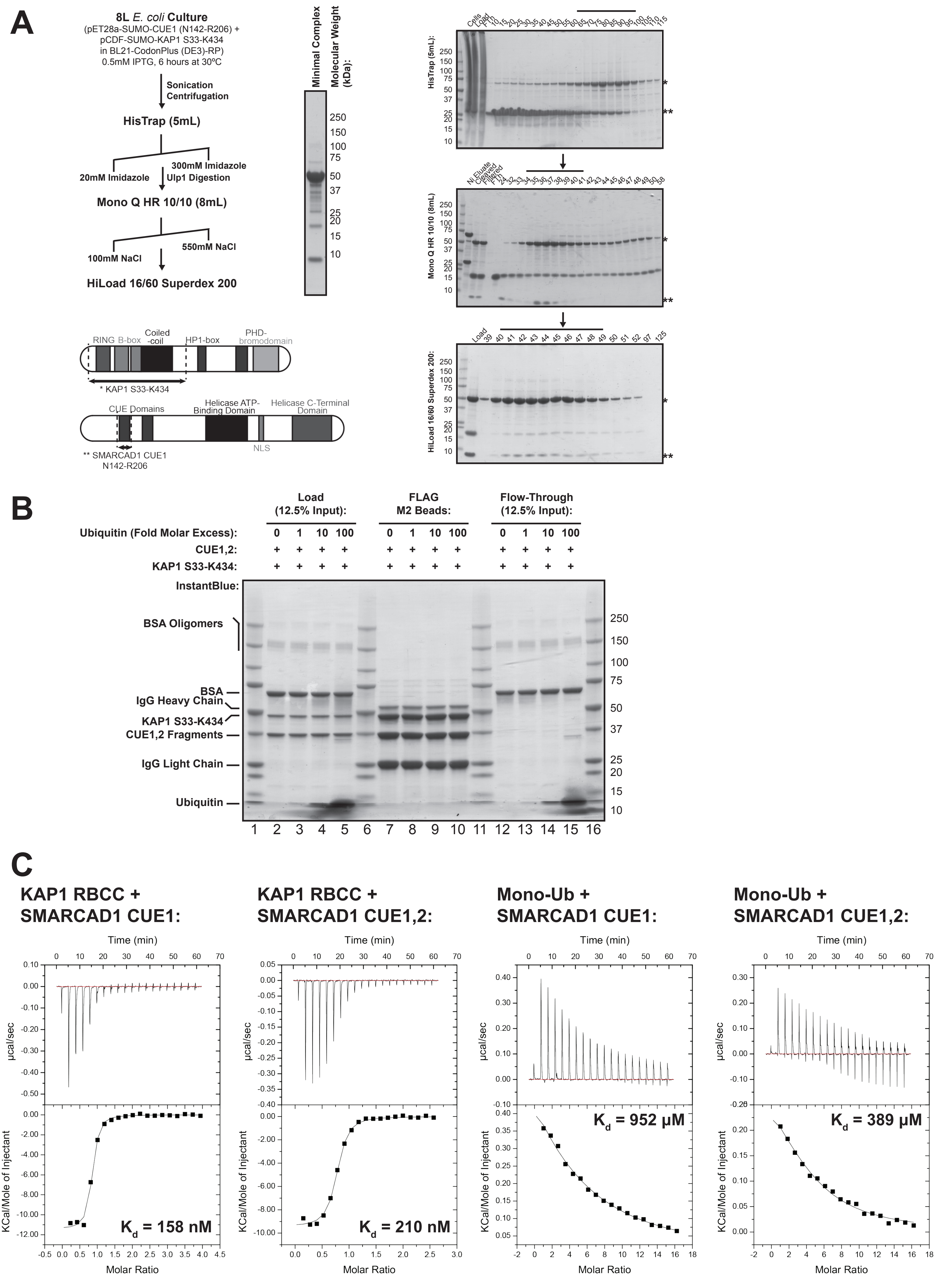

Figure S2, related to Figure 1

Figure S2, related to Figure 1. Biochemical and thermodynamic properties of the SMARCAD1-KAP1 interaction. A. Co-expression in *E. coli* of KAP1 RBCC S33-K434 (single asterisk) and the minimal SMARCAD1 CUE1 N142-R206 domain (double asterisks) resulted in the formation of a stable complex that co-eluted over multiple columns (right panels). **B.** A large molar excess of ubiquitin fails to outcompete binding of the KAP1 S33-K434 fragment to immobilized purified CUE1,2. **C.** ITC measurements of binding of either KAP1 RBCC (S33-K434) or mono-Ub, to SMARCAD1 CUE1 or CUE1,2. A representative data set is presented for each experiment, with raw data (top), integrated heat signals (bottom) and dissociation constants displayed. Thermodynamic parameters are shown in Table S1.

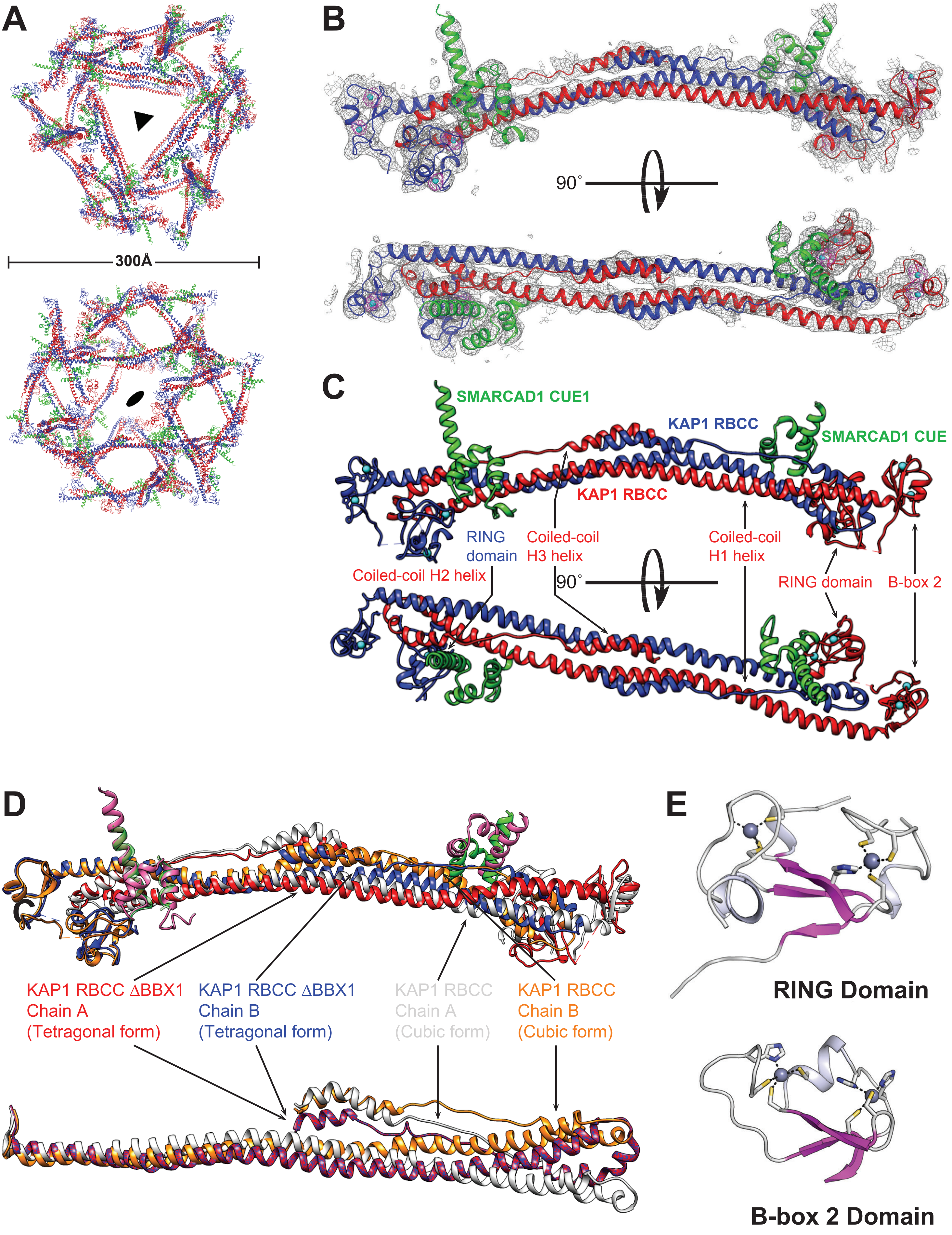

Figure S3, related to Figure 2

Figure S3, related to Figure 2. Structure of the KAP1 RBCC-SMARCAD1 CUE1,2 complex (cubic crystal form). A. The unit cell of the cubic crystal form is a proteinaceous cage with internal voids, with an extremely high solvent crystal content. **B.** Electron density map of the KAP1 RBCC-SMARCAD1 CUE1,2 complex (cubic form). One KAP1 RBBC chain is coloured red, the other blue, and the CUE1 domains green. Electron densities for the first B-box of KAP1 and the SMARCAD1 CUE2 are not seen. **C.** Structural model of the KAP1 RBCC-SMARCAD1 CUE1,2 complex, coloured as in Figure S3B. **D.** Comparison of the KAP1 RBCC ∆BBX1-SMARCAD1 CUE1,2 complex (tetragonal form; coloured red, blue & green) & KAP1 RBCC-SMARCAD1 CUE1,2 complex (cubic form; coloured grey, orange & pink). The KAP1 RBCC chains in the RBCC ∆BBX1 complex are symmetric, but slightly asymmetric in the RBCC complex, suggesting that the coiled coil domains may have a dynamic structure. **E.** The RING and B-box 2 domains of KAP1 are compact domains each consisting of a central three-stranded antiparallel β-sheet, short helix, several extended loops, and two coordinating zinc ions.

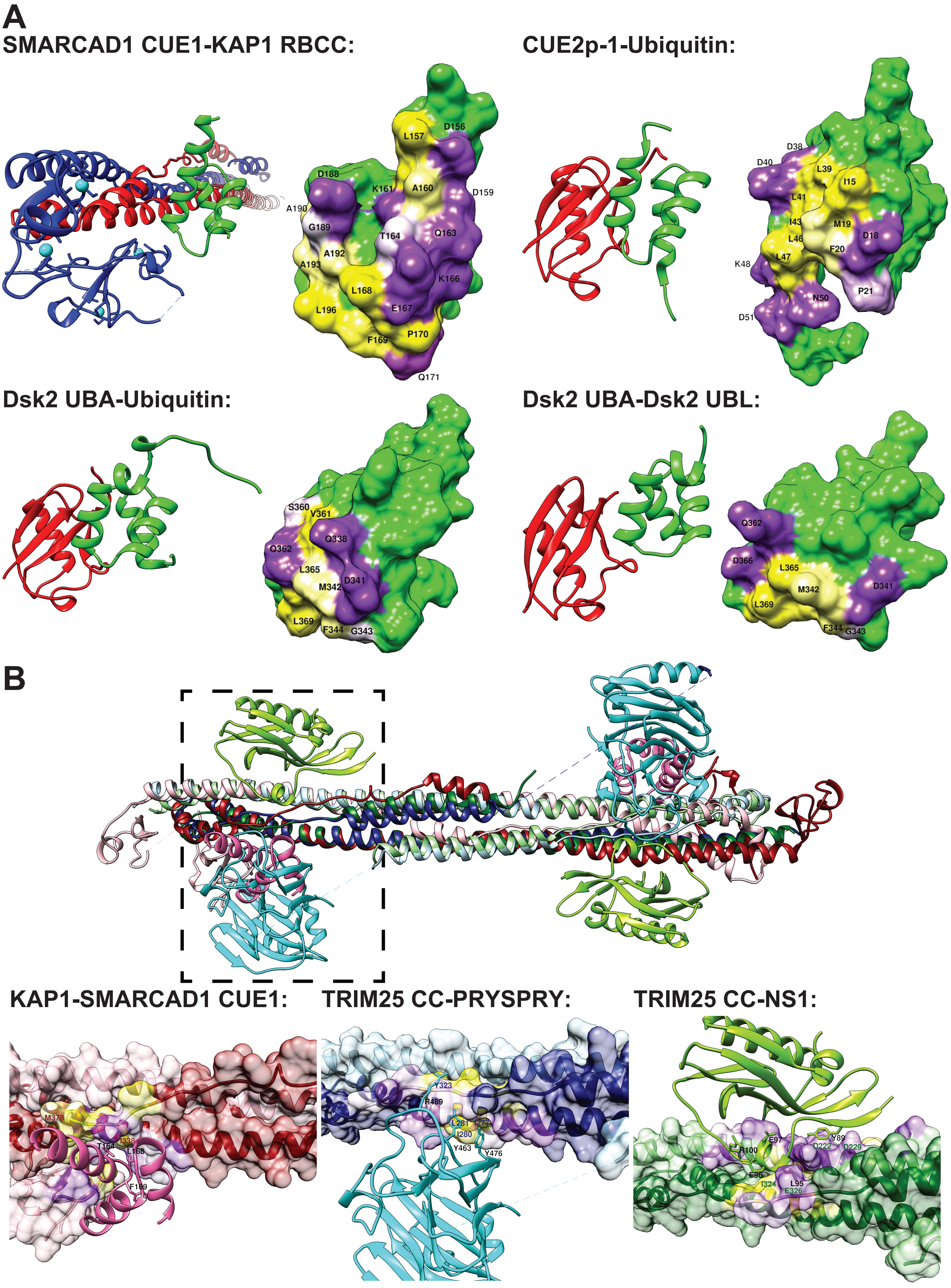

Figure S4, related to Figure 3

Figure S4, related to Figure 3. Comparative analysis of the KAP1 RBCC-SMARCAD1 CUE1 interaction surface. A. The surface employed by SMARCAD1 CUE1 to bind the KAP1 RBCC is comparable to that employed by other CUE and UBA domains for canonical ubiquitin or ubiquitin-like (UBL) domain recognition, in that they all rely upon the exposed surface formed by the α1 and α3 helices. The CUE and UBA domains are coloured green, whilst their ligands are red; the interaction surface is coloured by hydrophobicity as previously in Figures 2D and 3. **B.** Comparison of the KAP1 RBCC-SMARCAD1 CUE1 structure (chains coloured in shades of red) with TRIM25 coiled-coil in complex with the TRIM25 PRYSPRY domain (blue) or NS1 (green). The TRIM25 PRYSPRY and SMARCAD1 CUE1 domains bind to the same side, and to similar (but not identical) regions, of the coiled-coil domain. In contrast NS1 binds to the opposite side of the coiled-coil domain. The area enclosed by dotted lines is shown magnified in the images at the bottom, with the coiled-coil domains orientated comparably, and the interaction surface coloured by hydrophobicity.

Table S1. Thermodynamic parameters of binding of SMARCAD1 CUE1 or CUE1,2 to KAP1 RBCC and mono-ubiquitin. Related to Figure 1.

**Interaction**	**Temperature of Reaction (°C)**	K_d	**ΔH (kcal/mol)**	**-TΔS (kcal/mol)**
CUE1 + KAP1 RBCC	20	158 ± 35 nM	-11.9 ± 0.7	2.8 ± 0.7
CUE1,2 + KAP1 RBCC	20	210 ± 53 nM	-9.6 ± 0.2	0.6 ± 0.01
CUE1 + mono-Ub	10	952 ± 144 µM	1.2 ± 0.3	-5.1 ± 1.2
CUE1,2 + mono-Ub	10	389 ± 65 µM	0.4 ± 0.05	-4.8 ± 0.6

Table S2. Data collection and refinement statistics for the KAP1 RBCC ΔBBX1-SMARCAD1 CUE1,2 and KAP1 RBCC-SMARCAD1 CUE1,2 complexes. Related to Figure 2.

	**KAP1 RBCC-SMARCAD1 CUE1,2 (Cubic Crystal Form) (6H3A)**	**KAP1 RBCC ΔBBX1-SMARCAD1 CUE1,2 (Tetragonal Crystal Form) (6QU1)**
**Data collection statistics**		
Space group	I 2 3	P 4_1 2_1 2
Unit cell a, b, c, (Å)	299.9, 299.9, 299.9	64.5, 64.5, 287.9
Angles α, β, γ (°)	90, 90, 90	90, 90, 90
Wavelength (Å)	0.976	0.916
Resolution (Å)	80.1–5.50 (6.15–5.50)	29.4-3.70 (4.14-3.70)
*R*merge	0.155 (1.25)	0.223 (1.957)
*R*p.i.m.	0.04 (0.29)	0.106 (0.805)
I/σ*I*	11.5 (2.6)	7.3 (1.4)
CC1/2	0.999 (0.159)	0.997 (0.816)
Completeness (%)	100 (100)	99.7 (99.4)
Multiplicity	19.7 (19.5)	9.8 (7.3)
No. of unique reflections	14734 (3240)	7157 (1984)
**Refinement statistics**		
Resolution	75 - 5.5	29.4 – 3.70
*R*work/*R*free %)	27.7/30.3	29.7/34.5
**No. of atoms**		
Protein	5055	2440
Zinc ion	8	4
**Average B factors (Å^2)**		
All atoms	316	123
Protein	316	123
Ions	313	111
Wilson B	275	90
**Geometrical parameters**		
Bond lengths (Å)	0.004	0.003
Bond angles (°)	1.01	0.67
**Ramachandran plot statistics**		
Favoured (%)	88.66	93.4
Allowed (%)	10.1	5.3
Outliers (%)	1.24	1.3
